# Are stressful life events causally related to the severity of obsessive-compulsive symptoms? A monozygotic twin difference study

**DOI:** 10.1016/j.eurpsy.2014.11.008

**Published:** 2015-02

**Authors:** P. Vidal-Ribas, A. Stringaris, C. Rück, E. Serlachius, P. Lichtenstein, D. Mataix-Cols

**Affiliations:** a Department of Child and Adolescent Psychiatry, Institute of Psychiatry, King's College London, PO Box 85, 16, De Crespigny Park, London SE5 8AF, United Kingdom; b Department of Clinical Neuroscience, Division of Psychiatry, Karolinska Institutet, Stockholm, Sweden; c Department of Medical Epidemiology and Biostatistics, Karolinska Institutet, Stockholm, Sweden; d Department of Psychosis Studies, Institute of Psychiatry, King's College London, London, United Kingdom

**Keywords:** Twin study, Obsessive-compulsive symptoms, Life events, Abuse

## Abstract

Traumatic or stressful life events have long been hypothesized to play a role in causing or precipitating obsessive-compulsive symptoms but the impact of these environmental factors has rarely been investigated using genetically informative designs. We tested whether a wide range of retrospectively-reported stressful life events (SLEs) influence the lifetime presence and severity of obsessive-compulsive symptoms (OCS) in a large Swedish population-based cohort of 22,084 twins. Multiple regression models examined whether differences in SLEs within twin pairs were significantly associated with differences in OCS. In the entire sample (i.e., both monozygotic [MZ] and dizygotic twin pairs), two SLEs factors, “abuse and family disruption” and “sexual abuse”, were significantly associated with the severity of OCS even after controlling for depressive symptoms. Other SLEs factors were either not associated with OCS (“loss”, “non-sexual assault”) or were no longer associated with OCS after controlling for depression (“illness/injury”). Within MZ pair analyses, which effectively control for genetic and shared environmental effects, showed that only the “abuse and family disruption” factor remained independently related to within-pair differences in OCS severity, even after controlling for depressive symptoms. Despite being statistically significant, the magnitude of the associations was small; “abuse and family disruption” explained approximately 3% of the variance in OCS severity. We conclude that OCS are selectively associated with certain types of stressful life events. In particular, a history of interpersonal abuse, neglect and family disruption may make a modest but significant contribution to the severity of OCS. Further replication in longitudinal cohorts is essential before causality can be firmly established.

## 1 Introduction

Controlled family studies have consistently found that obsessive-compulsive disorder (OCD) is a familial disorder. For example, first-degree relatives of OCD probands are approximately 4 to 5 times more likely to have OCD themselves, compared to relatives of unaffected controls [53,69,44,52]. This familial risk decreases as the genetic distance with the proband increases. For example, second- and third-degree relatives are approximately 2 and 1.5 times more likely to have OCD, respectively, compared to relatives of unaffected controls, strongly suggesting a genetic component to OCD [44]. Twin studies primarily conducted in population-based (non-clinical) samples estimate the genetic contribution to be in the region of 40–50% [69,44,72]. Consistently, gene-searching efforts are well underway [67] and, though they have met with limited success to date, they are likely to continue [53].

Both family and twin studies also converge to suggest that shared environmental factors (e.g. growing up in the same neighbourhood) are unlikely to play a major role in causing OCD. Instead, the types of environmental influences that seem important in OCD are thought to be of the “non-shared” type, that is, environmental factors that affect specific individuals rather than families [72,28]. Such non-shared environmental factors could be biological (e.g. infections) or psychosocial (e.g. traumatic and stressful life events [SLEs], like being involved in an accident, being physically or sexually abused, or the death of one's partner). While the link between such SLEs and the pathogenesis of depression has been well-established [31,34], the evidence supporting a link between SLEs and OCD is much less conclusive because most studies have been conducted in clinical samples, based on retrospective reports and using designs that did not permit causal inferences. For example, research in clinical samples has shown that the association between OCD and post-traumatic stress disorder (PTSD) is larger than expected by chance [20,38]. One study [47] found that, compared to a control group, adults with OCD retrospectively-reported a greater number of SLEs one year before symptom onset. Similarly, in a large clinical sample of 265 adults with OCD, Cromer et al. [13] found that retrospectively-reported SLEs were associated with higher severity of OC symptoms (OCS) after controlling for a range of variables including depression severity. Similar findings have been reported in clinical samples of pediatric OCD [23,63]. However, clinical and convenience samples are liable to referral and Berkson's type biases, which may result in an overestimation of the associations.

A range of epidemiological, largely retrospective studies have also found associations between specific SLEs and OCD. For example, Mathews et al. [45] found an association between history of physical abuse, physical neglect and emotional abuse in childhood and OCS in a sample of 938 college students. Saunders et al. [64] and Boudreaux et al. [6] found that reported rape was associated with both lifetime and current OCD in a community sample of 391 women. Grisham et al. [24] found an association between retrospectively-reported sexual and physical abuse before age 11 and a diagnosis of OCD at ages 26 or 32, even after controlling for comorbid post-traumatic stress disorder. While, taken together, these findings are suggestive of an association between SLEs and OCD, they do not help clarifying the nature of this association. In particular, these studies could not control for genetic variability between individuals. This is particularly important as genetic background may not only put people at risk of developing psychopathology but also at risk of exposure to specific SLEs [30]. There is ample evidence that exposure to SLEs is at least partly heritable [30,56]. Therefore, isolating the non-shared variance of both OCS and SLEs is a crucial step to understand their association.

Genetically informative studies, in particular those employing the discordant monozygotic (MZ) twin design, are better suited to test whether the association between an environmental measure and an observed phenotype is likely to be consistent with a causal effect because they provide excellent control of both genetic and shared environmental effects. Because MZ-twins share 100% of their genetic background and grow up largely in the same environment, any observed phenotypic differences between members of a MZ-twin pair (e.g. differences in OCS) may be attributable to non-shared environment (e.g. non-shared SLEs) [73]. To our knowledge, only one previous study employed such design to test the association between SLEs and OCS in a population-based twin sample [12]. The authors selected 25 pairs of MZ-twins discordant for OCS (based on pre-determined cut-offs on a self-report questionnaire) and compared high vs low scoring twins on a number of potential environmental stressors, including retrospectively ascertained SLEs. They found suggestive evidence that SLEs, and particularly sexual assaults, were associated with OCS at the trend level (*P* = 0.08). It should be noted that this study did not adjust for other variables, such as depression, when comparing SLEs within discordant pairs. This is important because, not only there is a strong association between SLEs and depression, but also between depression and OCS [31,5,25]. Given that the evidence for the association between SLEs and OCS is only suggestive, it is plausible that part of this association might be explained by the presence of depressive symptoms. Thus, it is tenable that controlling for symptoms of depression may weaken the association between SLEs and OCS. Nevertheless, the suggestive results of Cath et al. study [12] merit replication in larger samples and expanding the number of SLEs examined.

In the present study, we used a genetically informative design to explore the relationship between a wide range of different SLEs and the severity of OCS in a large population-based sample of twins. Previous studies have often measured a limited range of different SLEs. In order to control for both genetic and shared environment effects and maximize the variance provided in our dataset, we employed the MZ-difference method [73,54]. This approach allows exploring whether the difference in SLEs between twins of a pair is associated with corresponding differences in the severity of OCS. This affords more statistical power than selecting high vs low symptom groups based on categorical cut-offs. Because there is a strong association between SLEs and depression and between depression and OCS [31,5,25], all our analyses controlled for depression severity in order to establish whether the presumed causal effect of SLEs on OCS is independent from depressive symptoms. Based on the previous literature, we made the following predictions:

• there will be a significant association between SLEs and OCS severity in the entire sample of twins (not controlling for genetic or family factors);
• SLEs will remain associated with OCS severity when genetic and shared environmental factors are strictly controlled in the analysis using the MZ-twin difference method;
• the association between SLEs and OCS severity will be generally weakened by the introduction of depression in the models as some of the variance may be explained by the known association between SLEs and depression.

## 2 Methods

### 2.1 Participants

Participants were drawn from the study of twin adults: genes and environment (STAGE), a study of 25,381 Swedish twins born between 1959 and 1985 [40]. Further details on the recruitment and data collection methods can be found elsewhere [40,16]. Briefly, twins were sent a letter inviting them to participate in the study and were given a personal login to the study web page. Non-responders were approached with up to three reminders. Twins could also choose to complete the questionnaire by telephone with a trained interviewer using a computer-based data collection method, supplemented with a mailed self-administered paper questionnaire for sensitive topics. Seventy-two percent of participants completed the web-based survey and 28% undertook the telephone interview. One hundred twins were re-contacted after 2 to 5 months in order to compare methods of data collection. For the OCD section, the agreement between the web-based survey and the telephone interview was perfect (Kappa = 1) [40]. The regional ethics committee of the Karolinska Institutet approved the project. All subjects provided informed consent electronically during the web-based survey or orally during the telephone interview.

Out of the original cohort, 22,517 twins completed a measure of OCS (see Measures section below). From these participants, we excluded all those who had at least one missing item on the OCS questionnaire. The final sample consisted of 22,084 twins (87% of the original cohort and 98% of the participants who completed the OCS questionnaire).

### 2.2 Measures

All participants completed the following self-report questionnaires as part of a larger wave of data collection.

#### 2.2.1 Stressful life events (SLEs)

Stressful life events were assessed with a modified version of the women, co-occurring disorders, and violence study (WCDVS) version of the life stressor checklist-revised (LSC-R) [46,75]. This adaptation showed to have acceptable test-retest reliability in all lifetime items as well as in the summary variables [46].

The instrument enquires about 34 different kinds of SLEs (e-Table 1, Supplementary data). The first 20 items, not included in the original LSC-R, enquire about general negative life events and are measured dichotomously (“yes” or “no”) depending on whether the event has happened to the respondent or not. The remaining 14 items, derived from the LSC-R, ask about interpersonal abuse and neglect. For each item, it asks about the occurrence (“yes” or “no”), the age when the event happened for the first time and its lifetime frequency (0 = never, 1 = once, 2 = a few times, 3 = a lot). For the purpose of this study, and in order to employ all 34 items, we only used information about the occurrence or non-occurrence of each event.

#### 2.2.2 Obsessive-compulsive symptoms (OCS)

A seven-item scale was employed to assess the lifetime presence and severity of the most common OCS (e-Table 2, Supplementary data). Each item is scored on a 3-point scale (0 = no, 1 = a little, 2 = a lot). This measure has been previously validated [44] in a clinical sample of 91 OCD patients who participated in a controlled clinical trial of internet-based cognitive behaviour therapy for OCD [3]. Briefly, the total score (sum of items 1–7) showed acceptable internal consistency (0.66) and correlated strongly with the criterion standards: Yale-Brown Obsessive-Compulsive Scale (YBOCS) [22]) total (Spearman's rho = 0.51), Obsessive-compulsive inventory-revised (OCI-R) [17] (rho = 0.76) and Dimensional Obsessive-Compulsive Scale (DOCS) [1] (rho = 0.66). These correlations are comparable than those reported in previous studies that employed alternative, more established self-report measures of OCS [12,14]. Conversely, the scale correlated more weakly and non-significantly with the Montgomery-Åsberg Depression Rating Scale (MADRS: [49]) (rho = 0.20, n.s.), indicating good discriminant validity.

#### 2.2.3 Depression

Because depression could confound the association between SLEs and OCS, we also included a measure of depression. We used the 11-item, Iowa version of the Center for Epidemiologic Studies Depression Scale [11]. This scale asks about depressive symptoms during the last 2 weeks, with the higher scores indicating higher depression severity. This shortened form of the original version of the CESD has proved to have robust psychometric properties, showing a high internal consistency (Cronbach's alpha ranged 0.71–0.88) and large correlations with the original version (range *r* = 0.81–0.93) in different populations [11,36].

### 2.3 Statistical analyses

#### 2.3.1 Data reduction

In order to reduce the number of SLEs items, we used a two-step factor analytic approach. First, in a random half of the sample, we used the 34 SLEs items in an Exploratory Factor Analysis (EFA) with categorical factor indicators using a promax rotation. This yielded 8 different factors (1 through to 8 factors) with eigenvalues higher than 1. Second, in the other random half of the sample, we used a confirmatory factor analysis (CFA) to compare between these 8 models after removing all non-loading items. We compared models using the Akaike Information Criteria (AIC) and the Bayesian Information Criteria (BIC), where lower values indicate better fit [39,9]. Goodness-of-fit was assessed in all models using Tucker Lewis Index (TLI), Comparative Fit Index (CFI) and Root Mean Square Error Of Approximation (RMSEA). For CFI and TLI, values greater than 0.95 are preferred and values near 0.90 are considered acceptable. For RMSEA, values of 0.05 or below are preferred and values up to 0.08 are considered acceptable [26]. Factor analyses were conducted in *M*plus, version 5.1 [50].

As shown in e-Table 3, Supplementary data, the best fit to the data was for a model with the following five factors: “illness/injury”, “abuse and family disruption”, “loss”, “sexual abuse” and “non-sexual assault”. The 5-factor model fitted the data better than a more parsimonious 4-factor model (Δ AIC 918.937, Δ BIC 890.416 and chi-square difference test: 6264.450, df = 9, *P* < 0.0001).

The CFA indicated that 28 of the 34 initial variables loaded clearly (> 0.32) and independently (difference > 0.20) onto one or two of the five factors (e-Table 1, Supplementary data) [68]. Items that loaded in two factors were Adoption (loading in factors “abuse and family disruption” and “loss”), emotional neglect (loading in factors “abuse and family disruption” and “sexual abuse”) and victim of a hate crime (loading in factors “sexual abuse” and “non-sexual assault”). Item of break-up with a friend strictly met criteria for being added in the “illness/injury” factor. However, this item was removed because it was thought to have no relation with the topic of the factor and the difference required to meet the independence criteria was in the lower bound (difference = 0.201). Finally, another six items were removed from the analysis because of low loadings on all factors. Thus, 27 items grouped in 5 factors were used in the analyses. Total scores for each factor were computed summing raw scores of individual items and standardizing them to *z*-scores to retain the scale metric. This has the same effect as using average scores, which are useful to do comparisons across factors when there are differing numbers of items per factor [15]. This approach is generally acceptable for most exploratory research situations [68]. It should be noted that 20% of the participants had missing SLEs data and they were thus excluded from the CFA.

#### 2.3.2 Phenotypic relation between SLEs and OCS

Multivariate linear regression was used in the whole sample to determine whether SLEs were associated with OCS severity. In a second step, we used current depressive symptoms as a covariate to explore whether the results persisted. Note that these analyses do not control for either shared environment or genetic effects. As twins of a pair cannot be considered fully independent observations, not controlling for this relation would lead to the overestimation of *P*-values. Therefore, we employed a robust cluster option to account for the non-independence of twin pairs, allowing the standard errors to correlate within-pairs.

#### 2.3.3 Within-pair analyses using the MZ-difference method

We analysed the relation between the lifetime occurrence of SLEs and the severity of OCS in all MZ complete pairs using within-pair difference scores in these variables [73,54]. The MZ-difference method uses relative difference scores, thus allowing for the examination of the association between SLEs and OCS severity with strict control of genetic and shared environmental factors. Relative difference scores were obtained by randomly assigning one of the twins as Twin 1 and the other as Twin 2, and subtracting the score of Twin 2 from the score of Twin 1. Multiple regression analyses were conducted to assess the contributions of differences in lifetime occurrence of SLEs and in current depressive symptoms between Twin 1 and Twin 2 to the prediction of differences in OCS severity between Twin 1 and Twin 2. Due to the high number of MZ-twin pairs with zero values in the OCS measure in both twins (57%), the distribution of differences in the OCS measure showed an excess positive kurtosis. Consequently, we decided to leave these pairs out of the analysis. As a result, 877 MZ-twin pairs were included in this analysis (819 pairs in the adjusted model).

All analyses described above were conducted in STATA 11 [65].

## 3 Results

### 3.1 Missing data analysis

In the entire sample, non-respondents to the OCS measure (*n* = 433) were more likely to be male (50% non-respondent vs 43% respondent; χ^2^ (1, *n* = 22,517) = 7.52, *P* < 0.01) and younger (32.4 vs 33.7 years old; *t* = 3.53, df = 22,515, *P* < 0.001) than respondents (*n* = 22,084). Non-respondents also reported higher depression severity than respondents (*t* = −2.96, df = 21,386, *P* < 0.01).

As mentioned earlier, of the 22,084 respondents to the OCS measure, around 20% had missing data on SLEs questions. People with missing SLEs data were more likely to be male (56% non-respondent vs 40% respondent; χ^2^ (1, *n* = 22,084) = [270.30, 386.26], *P* < 0.001), reported less severe OCS (*t* = [3.27,5.88], df = 22,082, *P* < 0.01) and more severe depressive symptoms (*t* = [−54.09, −51.18], df = 21,156, *P* < 0.0001).

Approximately 4% (*n* = 926) of the whole sample had missing data on depression severity. Non-respondents to the CESD were more likely to be female (57% respondent vs 61% of non-respondent females; χ^2^ (1, *n* = 22,084) = 7.67, *P* < 0.01) and reported higher OCS scores (*t* = −2.71, df = 22,082, *P* < 0.01).

Subsequent analyses were done with all the available data. Therefore, number of observations may vary across analyses.

### 3.2 Sample characteristics

Table 1 shows the sample composition by zygosity, and gender differences in age, OCS severity, depression severity (CESD score) and SLEs factors. In the whole sample, males were older than females. In addition, both in the entire sample and the MZ-twin subsample, females had significantly higher OCS scores and depression symptom severity. Whereas males reported more life events related to “illness/injury” and “non-sexual assault”, females were more likely to report life events related to “abuse and family disruption”, “loss”, and “sexual abuse”.

Regarding the association with age, younger participants scored higher in OCS and depression symptom severity, both in the whole sample (OCS: *r* = −0.10, 95% CI −0.11, −0.09, *P* < 0.0001; CESD score: *r* = −0.08, 95% CI −0.09, −0.06, *P* < 0.0001) and the MZ-twin subsample (OCS: *r* = −0.10, 95% CI −0.12, −0.07, *P* < 0.0001; CESD score: *r* = −0.09, 95% CI −0.11, −0.07, *P* < 0.0001).

Older participants in the whole sample reported more SLEs related to “illness/injury” (*r* = 0.04, 95% CI 0.03, 0.06, *P* < 0.0001), “loss” (*r* = −0.17, 95% CI 0.16, 0.19, *P* < 0.0001), and “non-sexual assault” (*r* = 0.02, 95% CI 0.001, 0.03, *P* < 0.05). In the MZ subsample, only “illness/injury” and “loss” were positively correlated with age (*r* = 0.04, 95% CI 0.02, 0.07, *P* < 0.001 and *r* = 0.15, 95% CI 0.12, 0.17, *P* < 0.0001, respectively).

Given these results, we decided to use gender and age as covariates in the subsequent analyses.

### 3.3 Phenotypic association between SLEs and OCS

As seen in Table 2, SLEs factors of “illness/injury”, “abuse and family disruption” and “sexual abuse” were all independently related to OCS severity in the whole sample. However, when adjusting for current depressive symptoms, only “abuse and family disruption” and “sexual abuse” remained significantly associated with OCS severity.

### 3.4 Within-pair analyses using a MZ-difference method controlling for genetic and shared environment effects

Table 3 shows that, when controlling for genetic and shared environment effects, only differences in the factor “abuse and family disruption” were positively and independently related to differences in OCS severity. Moreover, this association remained significant when adjusting for differences in current depressive symptoms. The factor “abuse and family disruption” explained 3% of the variance in OCS.

In order to get a deeper understanding of individual items contributing to these findings, we next performed paired *t*-tests within MZ-twins who were discordant for each individual item contained in this factor (Table 4). Monozygotic twins who reported exposure to “witnessing family violence before 18 years old”, “emotional neglect”, “physical neglect”, “physical abuse” and “serious family problem” scored higher in OCS severity than their respective unexposed twins. Conversely, there were no significant differences in OCS severity for the following two items: “separation or divorce of parents” and “having been adopted”.

## 4 Discussion

The aim of this study was to explore the nature of the known association between SLEs and OCS in a large population-based sample of twins. To this end, we employed a genetically informative design, the MZ-twin difference method, which allows a rigorous control of both genetic and shared environment factors. In addition, our models controlled for current depressive symptoms because OCS and depression often co-occur and there is a robust link between SLEs and depression. The results showed that there was a significant association between certain types of SLEs (history of physical abuse, neglect and family disruption) and OCS severity. Because this association remained significant when genetic factors, shared environmental factors and co-occurring depression were controlled for, we conclude that these SLEs could potentially play a modest (about 3% of the variance) but significant role in the severity of OCS. These findings, their implications and limitations of the study are discussed further below.

Our finding of a phenotypic association between certain types of SLEs and the severity of OCS in the entire sample (that is, when genetic and shared environmental factors are not controlled for) confirms our prediction and replicates the findings of numerous clinical and epidemiological studies where OCS were found to be associated to a wide range of SLEs, including physical and sexual abuse [13,45,64,6,24,66,8].

When we used the MZ-difference method to isolate the non-shared environment effect (i.e. controlling for genetic and shared environment effects), only SLEs related to “abuse and family disruption” were associated to differences in OCS severity. This association remained significant after controlling for depressive symptoms. This SLEs factor includes items such as “family violence in childhood”, “emotional abuse or neglect”, “physical neglect”, “physical abuse”, “serious family problem”, “separation or divorce of parents” and “having been adopted”. This finding is reminiscent of the literature on parenting styles in OCD. Indeed, this research suggests that some dysfunctional parenting styles could be associated to the severity of OCS. Specifically, high expressed emotion, parental over-control, parental over-protection, emotional rejection, and low emotional warmth have been previously linked to higher levels of OCS [74] and OCD [2]. However, we are unaware of studies directly investigating the link between SLEs and parenting styles in OCD.

It could be argued that the “abuse and family disruption” factor contains items that are, by definition, shared environment. Therefore, differences in this factor may simply reflect differences in recalling SLEs. When we examined individual items included in this factor, we found differences in OCS severity within MZ pairs discordant for “family violence in childhood”, “emotional abuse or neglect”, “physical neglect”, “physical abuse”, and “serious family problem”. Because STAGE twins are 19–47 years old, not all these items necessarily refer to one's early life. For example, the item “serious family problem” may involve difficulties within the individual's current family structure (e.g. with spouses or children). Likewise, the item “emotional abuse or neglect” not only involves the current family but also classmates and co-workers. On the other hand, items such as “family violence before 18” and “physical neglect” are more likely to have occurred in childhood and may therefore reflect “shared” experiences. However, it is important to note that even family factors traditionally considered as shared environment could technically be classified as non-shared (e.g., parenting being experienced as different among siblings) [55]. It is clear that prospective longitudinal studies are needed before these factors can be considered truly causal.

The significant association between sexual abuse and the severity of OCS lost its significance when genetic and shared environmental effects were controlled for. Similarly, Cath et al. [12] found a non-significant trend for higher rates of sexual assault in the high-scoring twins of MZ-discordant pairs for OCS. Together, these findings may suggest that perhaps the association between sexual abuse and OCS may be attributable to other mechanisms such as gene-environment correlations (i.e., a specific genetic liability also predispose to specific environments) or gene by environment interactions (i.e., specific genotypes can make individuals more or less reactive to different environments) [51]. These mechanisms have been shown to play a role in the association between SLEs and depression [33,32] and are also thought to be at play in OCD [44,70]. The precise genes that correlate or interact with certain types of SLEs to potentially cause OCS are not known but our findings delineate a clear avenue for future research in the sense that not all types of SLEs seem associated with the severity of OCS.

The SLEs factor we termed “illness/injury”, which includes items such as injury, physical illness or being involved in an accident, was also found to be associated with OCS severity but this association was no longer significant after controlling for depressive symptoms. Physical health and depression symptoms are strongly associated [19]. Likewise, strong associations exist between depression and OCD, with comorbidities ranging between 50–80% in adult samples [58,60]. Therefore, our results may suggest that SLEs related to physical health could be associated with greater depressive symptomatology (and vice versa), rather than being specifically associated with the severity of OCS. Alternatively, it could be that depression mediates the association between these SLEs and OCS severity. Longitudinal studies would be needed to examine the potential mediation effects of depression in the association between “illness/injury” SLEs and OCS.

It is interesting that other types of SLEs, such as the factors we termed “loss” (e.g. death of loved ones, loss of one's home) or “non-sexual assault” (e.g. being a victim of robbery or being physically attacked), were not significantly associated with the severity of OCS. The literature on depression and PTSD also reveals that interpersonal SLEs (i.e. physical abuse, emotional abuse, sexual abuse, etc.) but not other types of non-interpersonal SLEs (i.e. witnessing a crime, exposure to an accident or disaster, death of parent) are significantly associated with these disorders and their severity [42,18,27]. It has been suggested that exposure to interpersonal trauma as opposed to non-interpersonal trauma have an impact in affect self-regulation, biological self-regulation, self-perception and relationships in the future [18]. Regarding specific associations, emotional abuse has shown to be more strongly related to depressive disorders than anxiety disorders, whereas other types of interpersonal trauma, such as physical or sexual abuse, have shown a similar association with both anxiety and depressive disorders [27,21]. On a more detailed inspection of the OCD literature, it also appears that the most frequently mentioned SLEs are those relating to interpersonal events [45,6,24,41]. Non-interpersonal SLEs, such as birth of a child, arguments with family or change/difficulties at work/school have also been mentioned, albeit less frequently [4,59]. Few studies have tested interpersonal and non-interpersonal SLEs simultaneously [6,24,12]. It has been suggested that SLEs related to OCD/OCS are perceived as having more negative impact, being more undesirable and being more uncontrollable [47,23,37,35,71]. Also, it has been suggested that higher levels of “thought suppression” after a SLEs may be associated with higher levels of OCS [48]. Similar results have been reported in PTSD [10].

Current cognitive behavioural models of the disorder [57,61] have long postulated that certain “experiences” can result in the development of dysfunctional beliefs and maladaptive schemas about the environment and the self [62]. In turn, these beliefs are thought to have a small yet significant etiological role in OCD [70]. Our results shed some light on the possible types of “experiences” that may be etiologically implicated in the development of such dysfunctional beliefs and eventually result in distressing OCS. Precisely how these particular life experiences, and not other SLEs, result in OCD-related dysfunctional beliefs, OCS or both is unclear.

Although this study has a number of strengths, like the large twin sample and the broad coverage of interpersonal and non-interpersonal SLEs, some limitations should be noted. First, like in most previous studies, the assessment of SLEs was retrospective and recall biases cannot be fully ruled out. Moreover, as mentioned earlier, it is possible that individuals with greater OCS or depression severity may have a greater propensity to recalling certain types of SLEs. To explore this possibility further, we looked at the item “Did your parents ever separate or divorce while you were living with them?”. Although it is possible that parents got divorced when one of the twins in a pair was no longer living at home, eventually this item should be the same for both twins in a pair. Discordant MZ pairs on this item were almost 6% of pairs (134 pairs). There were no differences in depression scores or OCS severity between those twins who answered “yes” and those who answered “no” to this question. This means that divorce of parents has the same effect for both twins (whether they were living with them or not when they got divorced) or that, in case of recall bias, this is not affected by depression or OCS severity. However, we found differences in OCS severity within MZ-twin pairs discordant for other items included in the factor “abuse and family disruption”. Prospective and longitudinal studies are needed in order to establish which part of the variance in OCS is due to the causal effect of SLEs vs simply due to recall bias. One important limitation related to the cross-sectional design is that we lack information about the precise timing of SLEs and OCS and whether the former preceded the latter. Participants were asked for the time of occurrence of SLEs and the onset of OCS but, unfortunately, only 1.6% (range 0.1–5% depending on the SLE) had provided this information. A further limitation was that we only used information about the occurrence or non-occurrence of each SLE in order to employ all the possible types of life events. Information about the frequency or impact of SLEs might be of value for future studies, as the experience of a specific event may differ among individuals. The self-reported nature of the instruments and lack of corroborative evidence from multiple informants also potentially diminished the reliability of the reported information. The 7-item OCS measure is not a widely used instrument though its psychometric properties are remarkably sound [44]. In the future, these findings should be replicated employing longer and more extensively validated measures of OCS, such as the obsessive-compulsive inventory-revised [17] which may provide a broader range of scores. The use of such measures would also allow exploring whether specific associations exist between different SLEs and different OC symptom dimensions [43], in the same way as different types of SLEs seem to be related to different types of depressive symptom profiles [29]. Another possible limitation is that we could only adjust for current depressive symptoms, age and gender. Other variables, including personality and anxiety levels could be mediating the association between SLEs and OCS [23,45,7]. Finally, the generalizability of findings of this study may be somewhat reduced by the small but significant demographic and psychopathological differences between respondents and non-respondents to the questionnaires.

In conclusion, and with the above limitations in mind, this study suggests that certain types of stressful life events are selectively associated with the severity of OCS. In particular, a history of abuse, neglect and family disruption may make a modest but significant contribution to the severity of OCS. While the results are consistent with a causal interpretation, further replication in longitudinal cohorts is needed before causality can be confidently established.

## Disclosure of interest

The authors declare that they have no conflicts of interest concerning this article.

## Figures and Tables

**Table 1:** Sample characteristics and gender differences in OCS severity, depression severity and stressful life events (SLEs).

	All	Male	Female	Gender differences		
				*t*	df	*P*-value
*Total sample, n (%)*	22,084 (100)	9560 (43)	12,524 (57)	–	–	–
Complete pairs, *n* (%)	7127 (65)	2831 (62)	4296 (67)	–	–	–
Age, M (SD)	33.7 (7.7)	33.9 (7.7)	33.6 (7.6)	2.41	22,082	0.016
OCS, M (SD)	0.6 (1.4)	0.5 (1.2)	0.7 (1.5)	−11.14	22,082	< 0.0001
CESD, M (SD)	7.3 (5.5)	7.0 (5.1)	7.5 (5.9)	−6.13	21,156	< 0.0001
SLE factor score						
Illness/injury, M (SD)	1.2 (1.2)	1.3 (1.2)	1.1 (1.2)	14.02	17,782	< 0.0001
Abuse and fam. disrupt., M (SD)	1.3 (1.4)	1.1 (1.3)	1.3 (1.5)	−10.84	17,130	< 0.0001
Loss, M (SD)	1.1 (0.9)	1.0 (0.8)	1.2 (0.9)	−11.97	17,870	< 0.0001
Sexual abuse, M (SD)	0.7 (1.0)	0.4 (0.7)	0.9 (1.1)	−29.44	17,235	< 0.0001
Non-sexual assault, M (SD)	0.7 (1.0)	0.9 (1.1)	0.5 (0.8)	29.72	17,697	< 0.0001

*MZ-twins, n (%)*	8219 (37)^a^	3449 (42)	4770 (58)	–	–	–
Complete pairs, *n* (%)	2978 (72)	1127 (65)	1851 (78)	–	–	–
Age, M (SD)	32.4 (7.4)	32.6 (7.4)	32.3 (7.4)	1.51	8217	0.131
OCS, M (SD)	0.6 (1.4)	0.5 (1.1)	0.7 (1.6)	−8.67	8217	< 0.0001
CESD, M (SD)	7.3 (5.6)	6.9 (5.1)	7.5 (5.9)	−4.37	7885	< 0.0001
SLE factor score						
Illness/injury, M (SD)	1.2 (1.2)	1.3 (1.2)	1.1 (1.1)	8.18	6742	< 0.0001
Abuse and fam. disrupt., M (SD)	1.2 (1.4)	1.1 (1.3)	1.3 (1.5)	−5.74	6476	< 0.0001
Loss, M (SD)	1.1 (0.9)	1.0 (0.8)	1.1 (0.9)	−6.94	6772	< 0.0001
Sexual abuse, M (SD)	0.7 (1.0)	0.4 (0.7)	0.9 (1.1)	−18.12	6522	< 0.0001
Non-sexual assault, M (SD)	0.6 (1.0)	0.9 (1.2)	0.5 (0.8)	19.07	6694	< 0.0001
DZ twins, *n* (%)	13292 (60)^a^	5830 (44)	7462 (66)	–	–	–
Unknown zygosity, *n* (%)	573 (3)^a^	281 (49)	292 (51)	–	–	–

SLEs: stressful life events; SD: stress disorder; MZ: monozygotic. Ranges: age 19–47 years; OCS: obsessive-compulsive symptoms 0–14; CESD: Center For Epidemiologic Studies Depression Scale 0–33; illness/injury 0–4; abuse and family disruption 0–7; loss 0–6; sexual abuse 0–7; non-sexual assault 0–6.

a Percentage within the whole sample.

**Table 2:** Summary of multiple linear regression predicting the severity of obsessive-compulsive symptoms (OCS) from stressful life events in the whole sample of DZ and MZ-twins.

Stressful life events	OCS Severity (whole sample)			
	No adjustment for depression		With adjustment for depression	
	β	*t*	β	*t*
Factor 1: illness/injury	0.02	2.85^a^	0.01	1.36
Factor 2: abuse and family disruption	0.05	4.76^b^	0.03	2.85^a^
Factor 3: loss	−0.00	−0.07	0.00	0.06
Factor 4: sexual abuse	0.09	6.97^b^	0.07	5.76^b^
Factor 5: non-sexual assault	0.02	1.94	0.01	1.19
	Total *r* = 0.03 *F* (711,864) = 51.10 *P* < 0.0001		Total *r* = 0.05 *F* (811,574) = 54.57 *P* < 0.0001	

No adjustment for depression model, *n* = 16530; adjustment for depression model, *n* = 15919; β: standardised coefficient; analysis was done using robust clustering for twin pairs; all analyses are adjusted for age and gender.

a *P* < 0.01.

b *P* < 0.001.

**Table 3:** Summary of multiple regression analyses predicting within monozygotic pair differences in the severity of obsessive-compulsive symptoms (OCS) from within monozygotic pair differences in stressful life events.

Stressful life events	OCS Severity			
	No adjustment for depression		With adjustment for depression	
	β	*t*	β	*t*
Factor 1: illness/injury	−0.01	−0.31	−0.03	−0.99
Factor 2: abuse and family disruption	0.14	3.92^b^	0.11	3.26^a^
Factor 3: loss	−0.06	−1.73	−0.05	−1.47
Factor 4: sexual abuse	0.04	1.19	0.02	0.62
Factor 5: non-sexual assault	0.06	1.81	0.07	1.83
	Total *r* = 0.03 *F* (7869) = 3.89 *P* < 0.001		Total *r* = 0.06 *F* (8810) = 6.30 *P* < 0.0001	

No adjustment for depression model, *n* = 877 pairs; adjustment for depression model, *n* = 819 pairs; β: standardised coefficient; MZ-twin pairs where both twins scored zero on the OCS measure are excluded from the analyses; all measures are based on difference scores; all analyses are adjusted for age and gender.

a *P* < 0.01.

b *P* < 0.001.

**Table 4:** Difference in OCS severity between MZ-twin pairs discordant for individual items within the “Abuse and family disruption” factor.

Abuse and family disruption life event	Discordant pairs	OCS Severity M (SD)		Difference		
	*n*	Exposed twin	Unexposed twin	*t*	df	*P*-value
Family violence	362	0.98 (1.82)	0.70 (1.40)	2.97	361	0.003
Emotional neglect	749	0.82 (1.57)	0.65 (1.42)	2.71	748	0.007
Physical neglect	82	1.43 (2.14)	0.74 (1.22)	3.10	81	0.003
Physical abuse	363	0.95 (1.73)	0.72 (1.50)	2.62	362	0.009
Family problem	510	0.79 (1.68)	0.60 (1.27)	2.62	509	0.009
Parents divorced	134	0.56 (1.43)	0.60 (1.53)	−0.29	133	0.772
Adopted	32	0.84 (1.32)	1.25 (2.62)	−0.98	31	0.336

OCS: obsessive-compulsive symptoms; MZ: monozygotic; SD: stress disorder.
