# Erratum to “Are stressful life events causally related to the severity of obsessive-compulsive symptoms? A monozygotic twin difference study” [Eur. Psych. 30 (2015) 309–316]

**DOI:** 10.1016/j.eurpsy.2015.02.004

**Published:** 2015-07

**Authors:** P. Vidal-Ribas, A. Stringaris, C. Rück, E. Serlachius, P. Lichtenstein, D. Mataix-Cols

**Affiliations:** a Department of Child and Adolescent Psychiatry, Institute of Psychiatry, King's College London, London, United Kingdom; b Department of Clinical Neuroscience, Division of Psychiatry, Karolinska Institutet, Stockholm, Sweden; c Department of Medical Epidemiology and Biostatistics, Karolinska Institutet, Stockholm, Sweden; d Department of Psychosis Studies, Institute of Psychiatry, King's College London, London, United Kingdom

This article, as published in issue 2 of *European Psychiatry*, lacked necessary corrections due to a production error. The publisher regrets the mistake.

In the Methods section, the following studies and checklists should have been capitalized:

Study of Twin Adults: Genes and Environment (STAGE)
Women, Co-occurring Disorders, and Violence Study (WCDVS)
Life Stressor Checklist-Revised (LSC-R)
Obsessive-Compulsive Inventory-Revised (OCI-R)

The title of Table 1 should have read: Sample characteristics and gender differences in OCS severity, depression severity and stressful life events (SLEs).

The abbreviations DZ: dizygotic, and SD: standard deviation are here corrected.

In Tables 2 and 3: the *R*^*2*^ value was mistakenly noted as *r*.

