# Supplementary material for: Are stressful life events causally related to the severity of obsessive-compulsive symptoms? A monozygotic twin difference study
Source: Eur Psychiatry. 2015 Feb;30(2):309–16. doi: 10.1016/j.eurpsy.2014.11.008 (PMC4331096; doi:10.1016/j.eurpsy.2014.11.008)
Supplement: Supplementary file 1 [file mmc1.doc]

| **e-Table 1.** Five-Factor Model of Stressful Life Event Items. | | | | | |
| --- | --- | --- | --- | --- | --- |
| **Items** | **Illness/**  **Injury** | **Abuse and family disruption** | **Loss** | **Sexual abuse** | **Non-sexual assault** |
| Involved in an accident | **0.561** | -0.004 | 0.139 | -0.161 | 0.122 |
| Dropping a hobby, sport, craft, or recreational activity | **0.589** | -0.066 | -0.072 | 0.074 | 0.072 |
| Physical illness | **0.568** | 0.041 | 0.082 | 0.060 | -0.077 |
| Injury | **0.838** | 0.048 | -0.037 | -0.276 | 0.092 |
| When you were young before age 18, did you ever see physical violence between family members? For example, hitting, kicking or punching. | 0.102 | **0.935** | -0.144 | -0.094 | -0.020 |
| Have you ever been emotionally abused or neglected? For example, being frequently ashamed, embarrassed, ignored, or repeatedly told that you were no good. | 0.127 | **0.448** | -0.040 | **0.341** | -0.072 |
| Have you ever been physically neglected? For example, not fed, not properly clothed, or left to take care of yourself when you felt you were too young or ill. | 0.014 | **0.710** | 0.038 | 0.088 | 0.066 |
| Have you ever been physically abused - for example, hit, choked, burned, or beaten - or severely punished - for example, locked up, shut in a closet, tied up, or chained - by someone you knew well such as a parent, sibling, boyfriend or girlfriend? | -0.016 | **0.754** | -0.035 | 0.179 | -0.009 |
| Serious family problem | 0.113 | **0.390** | 0.185 | 0.180 | -0.050 |
| Did your parents ever separate or divorce while you were living with them? | -0.098 | **0.353** | 0.110 | -0.040 | 0.159 |
| Were you ever put in foster care or put up for adoption? | -0.111 | **0.419** | **0.407** | -0.095 | 0.188 |
| Death of spouse or partner | -0.234 | -0.080 | **0.570** | 0.192 | 0.054 |
| Death of a child | -0.092 | -0.013 | **0.447** | 0.018 | -0.001 |
| Death of other close family member | 0.191 | -0.007 | **0.567** | -0.017 | -0.256 |
| Destruction of home to natural disaster | 0.049 | -0.146 | **0.663** | -0.126 | 0.145 |
| Death of a close friend | 0.283 | -0.065 | **0.456** | 0.118 | -0.234 |
| Have you ever been discriminated against in a way that was highly distressing or disturbing because of your race, ethnic group, gender, sexual orientation, or religion? | 0.166 | -0.013 | -0.291 | **0.612** | 0.073 |
| Have you ever been bothered or harassed by sexual remarks, jokes, inappropriate touching, or demands for sexual favors by someone at work or school? For example, a co-worker, a boss, a customer, another student, or a teacher. | 0.069 | 0.054 | -0.077 | **0.666** | -0.152 |
| Were you ever touched or made to touch someone else in a sexual way, because you felt forced in some way or threatened by harm to yourself or someone else? | -0.133 | 0.033 | 0.114 | **0.885** | -0.111 |
| Did you ever have sex because you felt forced in some way or threatened by harm to yourself or someone else? By sex, I mean oral, anal, and/or genital. | -0.217 | -0.038 | 0.177 | **0.940** | -0.063 |
| Have you ever had sex when you did not want to in exchange for money, drugs, or other material goods such as shelter or clothing? | -0.136 | 0.057 | 0.106 | **0.540** | 0.248 |
| Have you ever been the victim of a hate crime? That is, have you ever experienced violence directed at you because of your race, ethnic group, gender, sexual orientation, or religion? | 0.127 | -0.126 | -0.282 | **0.552** | **0.406** |
| Involved in a lawsuit or court case | 0.086 | -0.015 | 0.230 | -0.011 | **0.522** |
| Have you ever been robbed, mugged, or physically, not sexually attacked by a stranger, or by someone you did not know well? | 0.100 | -0.009 | -0.058 | -0.206 | **0.729** |
| Have you ever seen a robbery, a mugging, or an attack taking place? | 0.051 | -0.018 | -0.035 | -0.076 | **0.646** |
| Have you ever been stalked, or has anyone ever threatened to kill you or seriously harm you? | -0.008 | 0.086 | 0.042 | 0.221 | **0.480** |
| Have you ever been strip searched, forcibly restrained, or held against your will by a provider of mental health or substance abuse services? | 0.001 | 0.131 | 0.041 | 0.139 | **0.351** |
| Loss of a job | 0.164 | -0.058 | 0.213 | 0.094 | 0.106 |
| Divorce or break-up with spouse or partner | 0.040 | 0.041 | 0.171 | 0.172 | 0.208 |
| Serious illness or injury of close family member | 0.157 | 0.001 | 0.330 | -0.022 | 0.084 |
| Problem with neighbours | 0.260 | 0.019 | 0.148 | 0.114 | 0.092 |
| Move to a worse residence or neighbourhood | 0.249 | 0.020 | 0.114 | 0.219 | 0.133 |
| Break-up with a friend | 0.396 | 0.101 | -0.010 | 0.195 | 0.042 |
| Financial loss | 0.290 | 0.005 | 0.295 | 0.052 | 0.276 |
| Note: Items were included if loadings on factors were >0.32 and the difference was >20. Items removed are at the bottom of the table. | | | | | |

| **e-Table 2.** OCD items from STAGE (each measured on a scale from 0=no, 1=a little, 2=a lot). | |
| --- | --- |
| 1. | Excessive cleaning: hand washing, baths, showers, tooth brushing, etc? |
| 2. | Other special measures to avoid dirt, germs or poisons? |
| 3. | Excessive checking: electric switches, gas taps, locks, doors, the oven? |
| 4. | Repeating the same simple activity many times in a row for no reason, e.g. repeatedly standing up or sitting down or going backwards and forwards through a doorway? |
| 5. | Touching things or people in particular ways? |
| 6. | Arranging things so they are just so, or exactly symmetrical? |
| 7. | Counting to particular lucky numbers or avoiding unlucky numbers? |

| **e-Table 3.** Confirmatory Factor Analysis for 5 different models of Stressful Life Events. | | | | | |
| --- | --- | --- | --- | --- | --- |
| Model | AIC | BIC | CFI | TLI | RMSEA |
| 4 Factors | 166447.650 | 166896.846 | 0.859 | 0.895 | 0.039 |
| 5 Factors | 165528.713 | 166006.430 | 0.921 | 0.941 | 0.029 |
| 6 Factors | 180982.721 | 181524.609 | 0.911 | 0.940 | 0.031 |
| 7 Factors | 173955.500 | 174511.648 | 0.933 | 0.956 | 0.027 |
| 8 Factors | 199037.480 | 199679.190 | 0.911 | 0.945 | 0.027 |
| CFI= Comparative Fit Index; TLI= Tucker-Lewis Index; RMSEA= Root Mean Square Error of Approximation; AIC= Akaike information Criteria; BIC= Bayesian Information Criteria.  Δ AIC and Δ BIC were calculated as the difference between a candidate model and the best (i.e. lowest AIC or BIC model). Values equal of greater to 10 for Δ AIC and Δ BIC indicate overwhelming support for the lower AIC and BIC models. | | | | | |
